# Real-world outcomes versus clinical trial results of immunotherapy in stage IV non-small cell lung cancer (NSCLC) in the Netherlands

**DOI:** 10.1038/s41598-021-85696-3

**Published:** 2021-03-18

**Authors:** Christine M. Cramer-van der Welle, Marjon V. Verschueren, Merel Tonn, Bas J. M. Peters, Franz M. N. H. Schramel, Olaf H. Klungel, Harry J. M. Groen, Ewoudt M. W. van de Garde, The Santeon NSCLC Study Group, E. A. Kastelijn, L. C. Vermeer, B. E. E. M. van den Borne, J. W. G. van Putten, J. H. Schouwink, A. A. J. Smit

**Affiliations:** 1 Santeon Hospital Group, Santeon, Herculesplein 38, 3584 AA Utrecht, The Netherlands; 2 Department of Clinical Pharmacy, St. Antonius Hospital, Utrecht, Nieuwegein, The Netherlands; 3 Department of Pulmonary Diseases, St. Antonius Hospital, Utrecht, Nieuwegein, The Netherlands; 4 Division of Pharmacoepidemiology and Clinical Pharmacology, Department of Pharmaceutical Sciences, Utrecht University, Utrecht, The Netherlands; 5 Department of Pulmonary Diseases, University Medical Center Groningen, University of Groningen, Groningen, The Netherlands; 6 Department of Pulmonary Diseases, Canisius-Wilhelmina Hospital, Nijmegen, The Netherlands; 7 Department of Pulmonary Diseases, Catharina Hospital, Eindhoven, The Netherlands; 8 Department of Pulmonary Diseases, Martini Hospital, Groningen, The Netherlands; 9 Department of Pulmonary Diseases, Medisch Spectrum Twente, Enschede, The Netherlands; 10 Department of Pulmonary Diseases, OLVG, Amsterdam, The Netherlands

**Keywords:** Non-small-cell lung cancer, Cancer immunotherapy, Outcomes research

## Abstract

This study aims to assess how clinical outcomes of immunotherapy in real-world (effectiveness) correspond to outcomes in clinical trials (efficacy) and to look into factors that might explain an efficacy-effectiveness (EE) gap. All patients diagnosed with stage IV non-small cell lung cancer (NSCLC) in 2015–2018 in six Dutch large teaching hospitals (Santeon network) were identified and followed-up from date of diagnosis until death or end of data collection. Progression-free survival (PFS) and overall survival (OS) from first-line (1L) pembrolizumab and second-line (2L) nivolumab were compared with clinical trial data by calculating hazard ratios (HRs). From 1950 diagnosed patients, 1005 (52%) started with any 1L treatment, of which 83 received pembrolizumab. Nivolumab was started as 2L treatment in 141 patients. For both settings, PFS times were comparable between real-world and trials (HR 1.08 (95% CI 0.75–1.55), and HR 0.91 (95% CI 0.74–1.14), respectively). OS was significantly shorter in real-world for 1L pembrolizumab (HR 1.55; 95% CI 1.07–2.25). Receiving subsequent lines of treatment was less frequent in real-world compared to trials. There is no EE gap for PFS from immunotherapy in patients with stage IV NSCLC. However, there is a gap in OS for 1L pembrolizumab. Fewer patients proceeding to a subsequent line of treatment in real-world could partly explain this.

## Introduction

For patients with stage IV non-small cell lung cancer (NSCLC), immune checkpoint inhibitors (ICIs) have demonstrated promising results in disease progression and survival in randomized clinical trials (RCTs)^1–6^. Currently, there are three FDA and EMA approved programmed cell death protein-1/programmed cell death ligand-1 (PD-1/PD-L1) inhibitors available for treatment of stage IV NSCLC. These include nivolumab (anti-PD-1), pembrolizumab (anti-PD-1), and atezolizumab (anti PD-L1)^1^. However, results from RCTs are not easily generalizable to clinical practice due to strict eligibility criteria^7–9^. While the population is ageing, which is associated with an increased risk of lung cancer, most patients that are included in RCTs are under 65 years old^10,11^. Additionally, NSCLC patients with comorbidities, especially auto-immune diseases, or poor performance status are often not eligible for participating in RCTs^11,12^. At present, RCTs are still the gold standard to prove efficacy for new oncology therapies^7,8^. Consequently, there could be a gap between the efficacy demonstrated in RCTs and the effectiveness of systemic therapies in clinical practice.

Recently, we have demonstrated that survival of patients with metastatic NSCLC treated with chemotherapy or targeted therapy is nearly one-quarter shorter in real-world practice than for patients included in trials^13^. For immunotherapy in metastatic NSCLC, some efforts have been made as well to provide insight into this possible gap. So far, most studies reported comparable outcomes in real-world compared to those observed in clinical trials. For example, a recent study from The Netherlands by Smit et al.^14^ assessed real-world effectiveness of ICIs in a Dutch population of patients with advanced NSCLC and showed similar median survival times as those of phase III studies, except for patients with Eastern Cooperative Oncology Group performance status (ECOG PS) of ≥ 2. The latter study, however, did not discriminate between type of drug nor compared survival curves to detect potential differences between survival dynamics in real-world and trials. Many studies have shown that the shape of the survival curve for immunotherapy is different than for chemotherapy, with some extra early deaths with immunotherapy and a plateau of long survivors later on. Furthermore, patient characteristics have not been compared with trial cohorts in search for explanatory factors for any efficacy-effectiveness gap.

The aim of this study is to assess how clinical outcomes of specific immunotherapies for patients with stage IV NSCLC in real-world (effectiveness) correspond to the results from clinical trials (efficacy) and to look into factors that might explain an efficacy-effectiveness gap.

## Patients and methods

### Data sources

Clinical data from six hospitals of the Santeon hospital network were used for this study. This hospital network consists of seven large (non-university) teaching hospitals that are geographically spread over The Netherlands and serve > 11% of the Dutch population ^15^. Patients diagnosed with stage IV NSCLC were identified from the Netherlands Cancer Registry (NCR)^16^. Data on diagnosis, vital status, and patient characteristics were obtained from the NCR.

Data on pharmacotherapy from the Santeon Farmadatabase was merged with the NCR dataset, to construct an overview of all applied regimes in the study population. The Santeon Farmadatabase contains, among others, information on individual prescriptions with drug name, dosage, date of administration and administration route^17^. Patients’ medical records were used to complement and validate the dataset.

Data was collected and managed with REDCap^18^, hosted at St. Antonius Hospital, Utrecht/Nieuwegein, the Netherlands.

### Study population

Patients with stage IV NSCLC diagnosed between January 1st, 2015 and December 31st, 2018 in one of six Santeon Hospitals were selected. Staging was based on the 7th edition TNM classification for the years 2015–2016 and 8th edition for incidence years 2017–2018. Characteristics that were recorded for included patients were age (at year of diagnosis), gender, ECOG PS, histology, brain metastases, pre-existing autoimmune disease, and PD-L1 expression of the tumour. Also, the type of treatment (best supportive care (BSC), chemotherapy, targeted therapy or immunotherapy), line of treatment, hospital where the patient was treated, and (in case of immunotherapy) information on immune related adverse events (irAE) and palliative radiotherapy were collected.

### Identification of systemic treatments per patient

First-line treatment was defined as the initial systemic treatment that was started after diagnosis. Second-line or further line treatment was defined as systemic treatment applied after completion or discontinuation because of disease progression of first or second-line treatment, respectively. After the identification of all different regimens we ordered patients in four different categories: chemotherapy, treatment with tyrosine kinase inhibitors (TKIs), immunotherapy, or best supportive care. For this study we focussed on immunotherapy and identified the most frequently used types of drugs (pembrolizumab and nivolumab) in first and second-line in our database. Second-line nivolumab for NSCLC in the Netherlands was introduced in March 2016. In 2017, first-line pembrolizumab for NSCLC with PD-L1 tumour proportion score (TPS) ≥ 50% was introduced.

### Reference outcome

After identification of the most commonly used types of immunotherapy, corresponding reference outcomes were established from clinical trials. These clinical trials were identified by a literature search on PubMed for clinical trials used for approval of immunotherapy drugs. If multiple registration studies were published with different patient populations, the study with the most comparable patient population (based on the distribution of stage, PD-L1 expression, and histology) to our cohort was chosen. We searched for updated publications of the selected studies (if applicable) to use up-to-date data and to utilize as much as possible of their follow-up time for the comparison with real-world data.

### Statistical analysis

All statistical analyses were conducted using R software package version 3.6.1.

To present an overview of baseline characteristics for all treated patients, frequencies (proportions) were calculated for categorical variables, and means (standard deviations) were provided for normally distributed continuous data.

For patients who received immunotherapy in first or second-line, overall survival (OS) was calculated as time between start date of treatment until date of death from any cause. Patients still alive at January 1, 2020 were censored as this was the end of follow-up date. Progression free survival (PFS) was calculated as time between start date of treatment until the occurrence of progression according to RECIST criteria when noted. Date of death was noted in absence of acknowledged progression from the individual patient files. Survival curves were obtained for the treatment groups using the Kaplan–Meier method.

The potential existence of an efficacy-effectiveness gap was assessed in two manners. First, a so-called efficacy-effectiveness (EE) factor was calculated by dividing the patient’s individual median survival by the corresponding reference OS and PFS from clinical trials^13^. This factor was used to estimate the presence of an efficacy-effectiveness gap and shows how the patient’s individual survival is related to survival presented in the reference RCT. As an example, an EE factor of 0.70 shows that median survival is 30% shorter in clinical practice than in RCTs. The Wilcoxon signed rank test was used to analyse the distribution of the EE factors. The null hypothesis (median OS in real-world is similar to median OS reported in clinical trials) is rejected if the distribution is significantly different from test value 1.0.

Second, we assessed hazard ratios (HRs) between real-world immunotherapy treatment regimens and groups in corresponding RCTs to compare OS and PFS. This was achieved by first digitizing the Kaplan–Meier (KM) curves for the immunotherapy arm from the included clinical trials with the R-package ‘digitize’. The extracted data points for survival probability were used for reconstructing the KM curve with the algorithm as described by Guyot et al.^19^ The coordinates of the KM curve from the published graph were read in with the R-coding script by Guyot et al., together with the information on numbers at risk and total number of events, to reconstruct the KM data. With this reconstructed individual patient data (IPD), KM curves and Cox HRs were estimated using the R routines ‘survfit’ and ‘coxph’.

Univariate and multivariate analyses for potential prognostic factors for PFS and OS in real-world were performed using Cox proportional hazards regression models. The factors with *p* < 0.05 on univariate analysis were included in the multivariate analysis. In this analysis, missing values were imputed by single stochastic regression imputation (single run with all available characteristics in the model). Pearson’s chi-square tests were performed to test if the proportion of the prognostic factor in real-world differed from reference RCT data, if applicable.

### Ethical statement

All methods were carried out in accordance with relevant guidelines and regulations. The study was approved by the Santeon institutional review board, and all clinical information was provided in a de-identified fashion and informed consent was waived (SDB219-008). The study was performed in accordance with the ethical standards of the institutional and national research committee and with the 1964 Helsinki declaration and its later amendments or comparable ethical standards.

## Results

### Baseline characteristics

**Figure 1:**
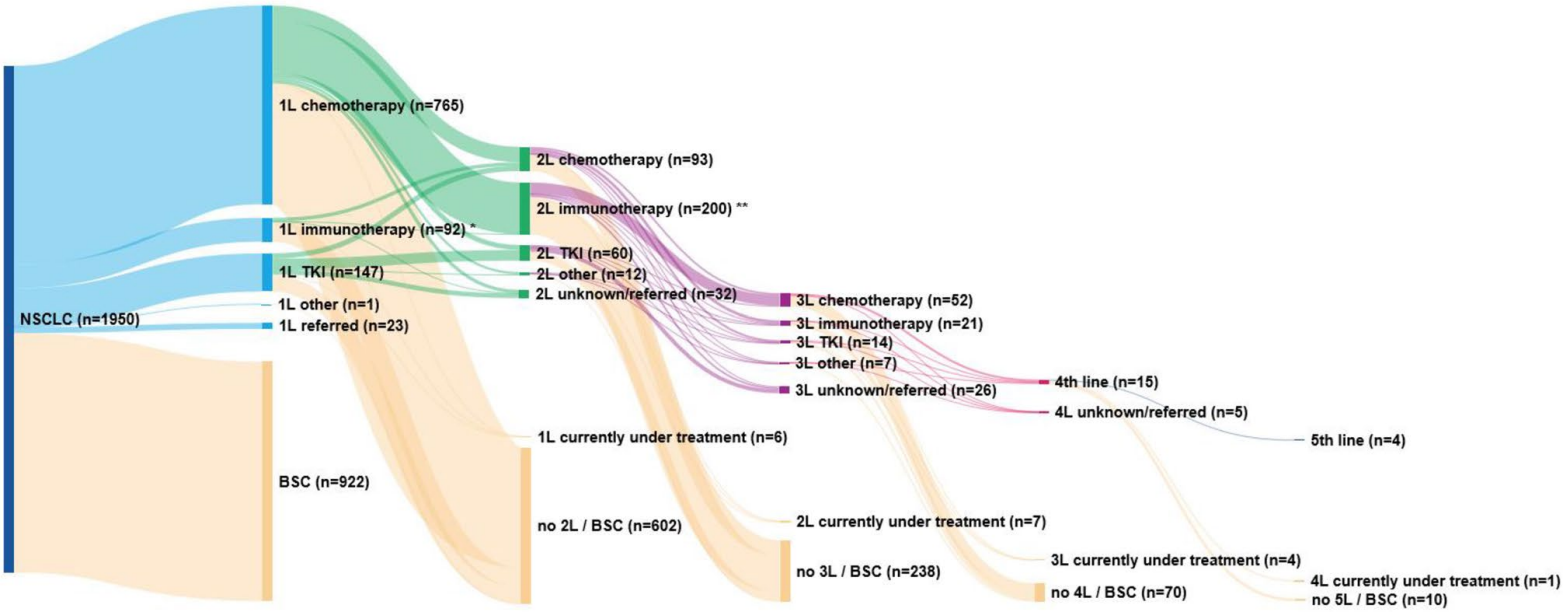
Treatment patterns of patients diagnosed with stage IV NSCLC between 2015 and 2018 in six Dutch hospitals. BSC, best supportive care; TKI, Tyrosine Kinase Inhibitors. *1L immunotherapy: pembrolizumab: n = 83 (90%), nivolumab: n = 3 (3%), other n = 6 (7%). **2L immunotherapy: nivolumab: n = 164 (82%, of which n = 141 with non-squamous histology), pembrolizumab: n = 20 (10%), atezolizumab: n = 13 (6.5%), other: n = 3 (1.5%).

We identified 1950 patients diagnosed with stage IV NSCLC in the period 2015 to 2018. Figure 1 provides an overview of the different treatment patterns of all patients towards best supportive care (BSC). Of these patients, 922 (47%) did not receive active anti-tumour treatment in 1L because of their ECOG PS, comorbidities, or at request of the patient. Twenty-three patients (1%) were referred to other hospitals after diagnosis of whom we did not have information on treatment. Of all diagnosed patients, 1005 (52%) received 1L treatment of which 92 (9%) received immunotherapy in first-line. Median OS for patients receiving chemotherapy, immunotherapy or TKI was 7.5 months, 15.6 months or 15.5 months, respectively (Appendix SFigure 1).

Of all treated patients, only 365 (36%) received subsequent treatment in one of the six hospitals of which 200 (55%) received immunotherapy. The most frequently received 1L immunotherapy was pembrolizumab (n = 83, 90% of all patients treated with immunotherapy in 1L) and in second-line nivolumab for patients with non-squamous tumour histology (n = 141, 71%). Based on these subgroups, two registration studies (Checkmate 057 and KEYNOTE-024 ^2,5^) were identified for the comparison with real-world data.

Table 1 shows patient characteristics of patients with 1L pembrolizumab or 2L nivolumab (both monotherapy). The median age of patients with 1L pembrolizumab was 66 years and almost all patients (96%) had an ECOG PS of 0 or 1. The median age of patients with 2L nivolumab was 64 years and 95% had an ECOG PS of 0 or 1.

**Table 1:** Patient characteristics of stage IV NSCLC patients with 1L pembrolizumab or 2L nivolumab.

	Pembrolizumab 1st line		Nivolumab 2nd line	
	Real-world	RCT^5^	Real-world	RCT^2^
Subjects	83	154	141	292
Male	45 (54%)	92 (60%)	74 (53%)	151 (52%)
Age (median (range))	66.0 (35–84)	64.5 (33–90)	64.0 (44–80)	61.0 (37–84)
**ECOG PS**				
0	30 (36%)	54 (35%)	44 (31%)	84 (29%)
1	50 (60%)	99 (64%)	90 (64%)	208 (71%)
2	3 (4%)	1 (1%)	7 (5%)	–
**Histology**				
Squamous	11 (13)	29 (19%)	–	–
Non-squamous	72 (87)	125 (81%)	141 (100%)	292 (100.0)
**PD-L1 expression**				
< 1%	–	–	33 (23%)	108 (37%)
1–49%	–	–	29 (21%)	123* (42%)
≥ 50%	83 (100%)	154 (100%)	10 (7%)	
Unknown***	–	–	69 (49%)	61 (21%)
CNS metastases	16 (19%)	18 (12%)	25 (18%)	34 (12%)
Subsequent systemic therapy	14 (17%)	56 (36%)**	40 (28%)	123 (42%)

ECOG PS, Eastern Cooperative Oncology Group Performance Status; CNS, central nervous system; RCT, randomized clinical trial.

*PD-L1 expression of ≥ 1%.

**Including surgery and radiation therapy.

***Not performed in real-world; not quantifiable from RCT^2^.

### Survival

Median PFS and OS for 1L pembrolizumab treatment were 8.9 and 15.8 months, respectively. For patients treated with 2L nivolumab, median PFS was 3.8 and median OS was 8.2 months (Table 2). One-year survival was 57% for 1L pembrolizumab and 42% for 2L nivolumab. In the corresponding clinical trials on pembrolizumab and nivolumab, one-year survival was 70% and 51%, respectively^2,20^.

**Table 2:** Median survival in real-world clinical practice and randomized clinical trials.

	Real-world months (95% CI)	Clinical trial months (95% CI)	EE factor median
**1L Pembrolizumab**			
mPFS	8.9 (3.7–14.1)	10.3 (6.7–NR)^5^	0.85
mOS	15.8 (9.4–22.1)	30.0 (18.3–NR)^33^	0.45^a^
**2L Nivolumab**			
mPFS	3.8 (3.0–4.7)	2.3 (2.2–3.4)^34^	1.61^a^
mOS	8.2 (5.9–10.6)	12.2 (9.7–15.1)^34^	0.65

mPFS, median progression-free survival; mOS, median overall survival; EE factor, efficacy-effectiveness factor.

^a^Significantly different (*p*-values < 0.05) from test value 1.00 (one-sample Wilcoxon signed-rank test).

### EE factor analysis

Median OS was shorter for all patients who received 1L pembrolizumab in real-world practice compared to the clinical trial. Table 2 shows an EE factor of 0.45 (*p* < 0.001 from 1), which means that median survival is 55% shorter for patients treated in clinical practice relative to median survival from the registration clinical trial. There was no significant difference in median PFS for 1L pembrolizumab, with an EE factor of 0.85 (*p* = 0.86).

For 2L nivolumab, the EE factor for median OS is 0.65 (*p* = 0.065). For PFS, we found an EE factor of 1.61 (*p* < 0.001) (Table 2). This indicates that median PFS is 61% higher for nivolumab in clinical practice, compared to median PFS from the RCT.

### Proportional hazard analysis

**Figure 2:**
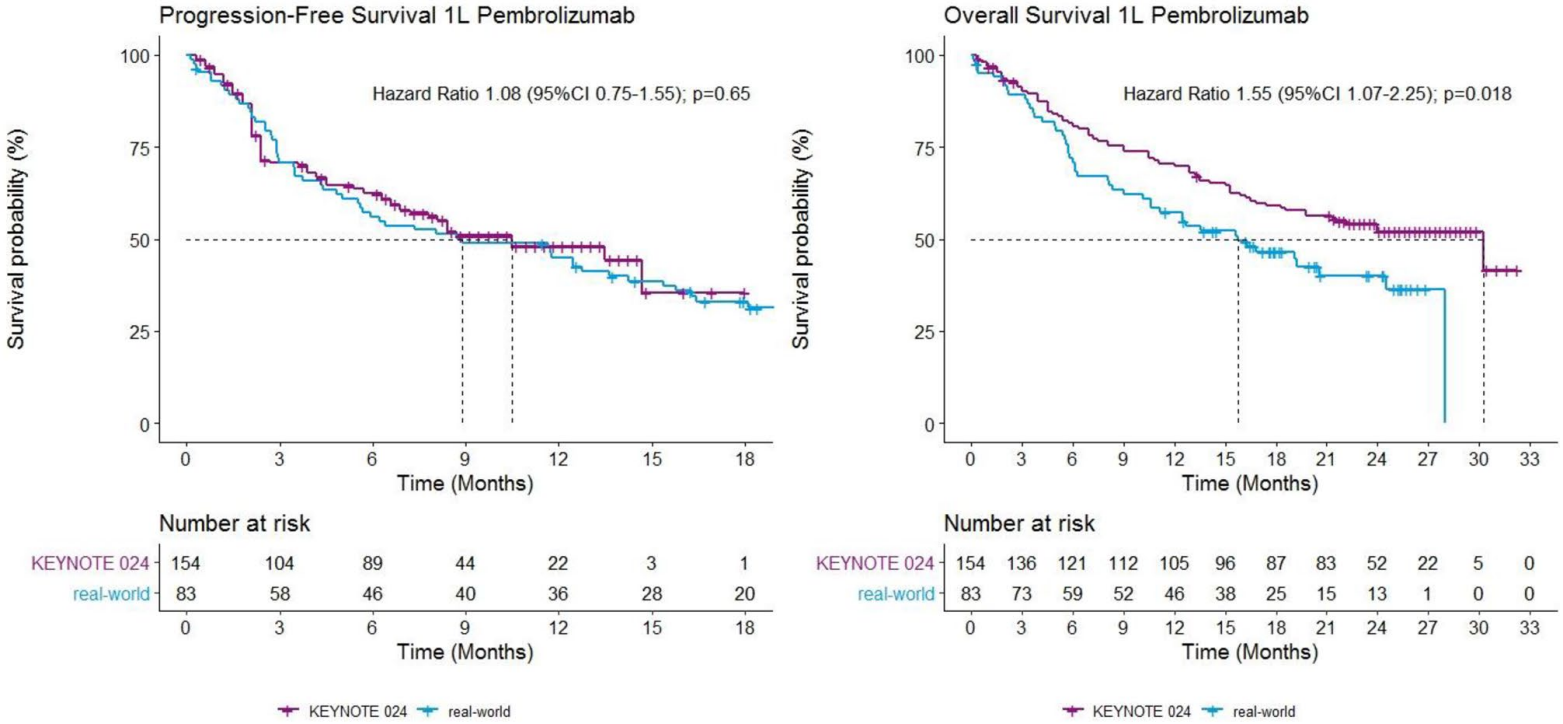
Kaplan–Meier curves of PFS and OS in patients receiving 1L pembrolizumab in real-world and clinical trial.

**Figure 3:**
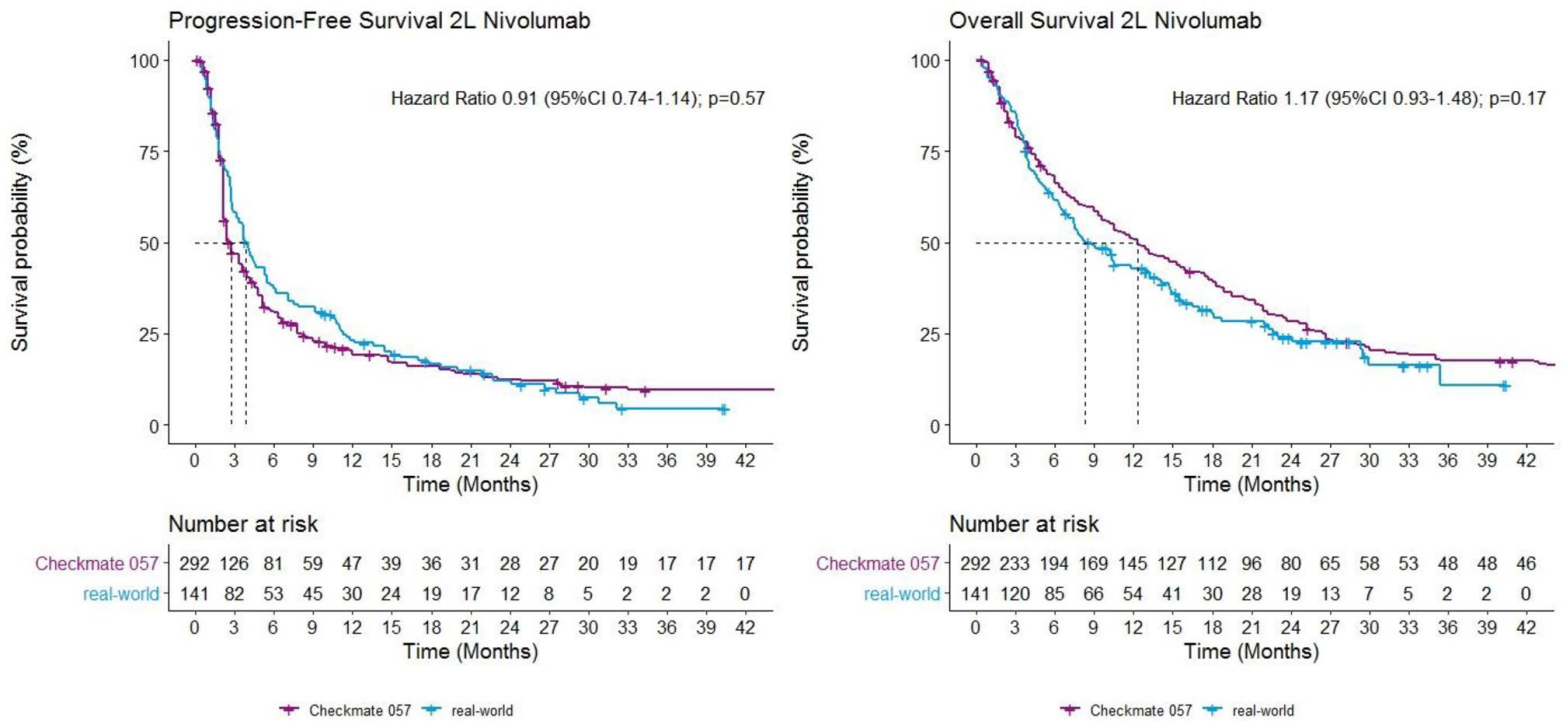
Kaplan–Meier curves of PFS and OS in patients receiving 2L nivolumab in real-world and clinical trial.

The estimated HRs for 1L pembrolizumab in real-world compared to data from the RCT were 1.08 (95% CI 0.75–1.55) and 1.55 (95% CI 1.07–2.25) for PFS and OS, respectively (Fig. 2). For 2L nivolumab the estimated HRs were 0.91 (95% CI 0.74–1.14) for PFS and 1.17 (95% CI 0.93–1.48) for OS (Fig. 3). This indicates that PFS is comparable between real-world and RCTs, and in real-world OS is significantly shorter for 1L immunotherapy.

From the univariate cox proportional hazards regression model, no significant associations were found with PFS or OS for all potential prognostic factors in real-world patients with 1L pembrolizumab, or for PFS in real-world patients with 2L nivolumab. Higher ECOG PS (0–1 vs. ≥ 2) at start of 2L nivolumab was associated with shorter OS (HR 2.23; 95% CI 1.03–4.83; *p* = 0.043). Further analysis showed that the proportion of patients with ECOG PS ≥ 2 in real-world is higher compared to patients in clinical trials (5% versus 0%, *p* < 0.001).

When looking at explanatory factors after starting 1L pembrolizumab in real-world, we found that PFS was influenced by the occurrence of irAEs. Having an irAE reduces the hazard by a factor of 0.48 (95% CI 0.26–0.89; *p* = 0.019). For 2L nivolumab, the occurrence of irAEs was also an explanatory factor for longer PFS and OS (HR 0.45; 95% CI 0.30–68; *p* < 0.001 and HR 0.44; 95% CI 0.27–0.70; *p* < 0.001, respectively).

In real-world, the proportion of patients with irAEs was significantly lower compared to clinical trials for both 1L pembrolizumab and 2L nivolumab (31% versus 77% with *p* < 0.001, and 28% versus 69% with *p* < 0.001, respectively). Furthermore, fewer patients received a subsequent line of treatment in real-world compared to patients in clinical trials after both 1L pembrolizumab and 2L nivolumab (17% versus 36% with *p* = 0.002, and 28% versus 42% with *p* = 0.014, respectively).

## Discussion

This study showed that PFS of patients with stage IV NSCLC treated with immunotherapy is comparable between real-world and clinical trials. However, OS is significantly shorter for patients with 1L pembrolizumab in real-world (median 15.8 vs. 30 months; HR 1.55 (95% CI 1.07–2.25)). This finding is in line with our previous research^13^ which showed that survival after first-line chemotherapy and targeted therapy is around one quarter shorter in real-world compared to clinical trial data. The present study extends this finding to first-line pembrolizumab monotherapy in patients with ≥ 50% PD-L1 expression.

To our knowledge, this is the first study that calculated hazard ratios for PFS and OS of real-world versus clinical trials. This approach provided insight that stage IV NSCLC patients who are treated with immunotherapy in regular clinical practice have a comparable period of PFS time but that this does not extend to a similar OS benefit as demonstrated in the registration trial. This implies that something differs between regular practice and clinical trial participants after progression on immunotherapy. One explanation from our data could be the receiving of a next line of treatment being more frequent in clinical trial participants compared to in real-world (two times more frequent in 1L setting, and 1.5 times in 2L setting). Patients who received immunotherapy through participating in a clinical trial, for example, might have an above average intrinsic willingness to search for further treatment options after treatment failure. Other possible explanations could be that clinics active in trial enrolment communicate more about remaining experimental treatments, or that patients characteristics, other than performance status, limit the tolerability for subsequent systemic treatment (in this case chemotherapy) after progression on immunotherapy to a larger extent in regular care patients.

The results on median OS and PFS as found in our study are largely in line with previous observational studies on 1L immunotherapy treatment with pembrolizumab in real-world. Velcheti et al.^20^ evaluated real-world survival in patients with metastatic NSCLC with an PD-L1 expression of ≥ 50% and ECOG PS ≤ 1, and found a median PFS of 6.8 months and median OS of 19.1 months. Their PFS and OS are slightly shorter than found in the KEYNOTE-024 clinical trial, but real-world OS is longer than was found in our study, which could be explained by only including patients with ECOG PS 0 or 1, or by the higher percentage of patients with second-line systemic therapy as compared to our cohort (28% vs. 17%, respectively). A French study which included patients with advanced NSCLC and PD-L1 expression of ≥ 50%, reported a median PFS of 10.1 months and a median OS of 15.2 months for patients treated with pembrolizumab in first-line, including patients with brain metastases and ECOG PS 2^21^. As in the results of the current study, their PFS is comparable with the median PFS from the KEYNOTE-024 trial of 10.3 months, whereas OS is shorter (comparable to our real-world findings). Tambo et al.^22^ found a median of 6.1 for PFS which is shorter than the median PFS from both the registration study and the current study. Shorter PFS may be explained by inclusion of patients with ECOG PS 2 (12%) and 3–4 (11%). Their OS did not reach the median and therefore could not be compared.

The outcomes found for 2L nivolumab are also in line with previous studies with real-world data. Crinò et al.^23^ found a median PFS for patients treated with nivolumab in second or further line of 4.2 months and a median OS of 7.9 months, which is comparable with the present findings (3.8 and 8.2 months, respectively). Another study in Dutch patients treated with second-line nivolumab reported a median PFS and OS of 2.6 and 10.0 months, respectively^24^. Grossi et al.^25^ reported results from the expanded access program in Italy on 1588 patients, including patients with ECOG PS 2 and aged ≥ 75 years, where median PFS was 3.0 months and median OS 11.3 months. The results of those three studies on real-world nivolumab in second-line suggest that results for PFS and OS in real-world and RCTs are indeed comparable. Interesting from our data is that the median PFS from 2L nivolumab was significantly longer than in the clinical trial but without any difference in overall hazard (HR 0.91). An explanation could be a difference in how date of progression is determined between the two settings. To illustrate, in Checkmate 057 disease progression was assessed nine weeks after start of treatment and every six weeks thereafter, compared to every 8 weeks in real-world. This relatively small difference in timing could lead to larger absolute differences with a median PFS in weeks range in 2L settings. Besides this, our finding also highlights that comparing median PFS times only could lead to biased conclusions about relative effectiveness.

Regarding prognostic factors, for patients treated with 2L nivolumab, the negative association between a higher/worse ECOG PS and OS is in line with previous research. Crinò et al.^23^ also found ECOG PS to be a poor prognostic factor. Additionally, the results from Schouten et al.^24^ from the Dutch expanded access program and the study by Martin et al.^26^ show similar results. A Dutch study on real-world effectiveness of ICIs in patients with stage IV NSCLC in first and second-line also shows that an ECOG PS ≥ 2 is a poor prognostic factor^14^. Additionally, in a phase 3B/4 community-based study of nivolumab monotherapy in previously treated patients with advanced NSCLC including patients with poor performance status (Checkmate 153), a median overall survival of 4.0 months was found in patients with ECOG PS 2^27^. Next to the association of ECOG PS ≥ 2 and worse outcome for patients with 2L nivolumab in real-world, the proportion of patients with ECOG PS ≥ 2 in real-world is significantly higher compared to the clinical trial. This confirms the general thinking that trials select more fit patients, where patients with higher ECOG PS in clinical practice aim for immunotherapy as well, resulting in an EE gap.

Besides this, our data also confirms the relationship between irAEs and outcomes of immunotherapy. Similar to Lisberg et al.^28^ who retrospectively analysed the relationship in patients that were treated with pembrolizumab in KEYNOTE-001, we found improved survival for patients with irAEs. Another study by Haratani et al.^29^ also revealed that irAEs were positively associated with survival outcome in nivolumab treated patients. Ricciuti et al.^30^ confirmed the positive relationship between the occurrence of irAEs and survival for patients with 2L nivolumab as well.

The main strengths of this study are its complete and precise data, nationwide multicentre approach, and that PFS and OS were compared both through comparison of medians as well as through a proportional hazards approach. The latter method better addresses that many patients were still alive at time of conducting the analyses and provides a unique opportunity to compare survival dynamics not captured by a single value for median survival.

Limitations of this study are that (patient) factors potentially responsible for an efficacy-effectiveness gap could only be analysed on the cohort level instead of patient level, because individual patient data from the respective clinical trials were not available. When available, these could have been analysed in a multivariable cox regression model potentially leading to identification of additional factors associated with the EE gap. Better identification of these factors could lead to trial designs matching real-world populations better and eventually smaller EE gaps. Besides this, another limitation is that the treatment patterns shown in Fig. 1 do not match with current practice anymore (introduction of 1L immunochemotherapy combination limiting the use of 2L immunotherapy). Nevertheless, this does not compromise the relative effectiveness assessments within specific treatments as conducted in our study.

Apart from the well-known complexity of how to translate progression free survival times to overall survival when survival data of the trial cohort is not mature yet^31,32^, the present study shows that this translation can be even more complex later on when it comes to generalizability to real-world settings. Considering that cost-effectiveness assessments are often based on overall survival data from trials, our observed shorter overall survival time after 1L pembrolizumab in real-world compared to the clinical trial data could provide an argument to rework the initial cost-effectiveness assessment.

## Conclusion

There is no efficacy-effectiveness gap for the outcome PFS for immunotherapy in patients with stage IV NSCLC. However, there is a gap in OS for first-line pembrolizumab. Fewer patients proceeding to a subsequent line of treatment in real-world could partly explain this. PFS and OS results from clinical trials can differ in generalizability to regular clinical practice.

## Supplementary Information

Supplementary Information
