# Supplementary material for: Real-world outcomes versus clinical trial results of immunotherapy in stage IV non-small cell lung cancer (NSCLC) in the Netherlands
Source: Sci Rep. 2021 Mar 18;11:6306. doi: 10.1038/s41598-021-85696-3 (PMC7973789; doi:10.1038/s41598-021-85696-3)
Supplement: Supplementary file 1 — Supplementary Information [file 41598_2021_85696_MOESM1_ESM.docx]

**APPENDIX to “Real-world outcomes versus clinical trial results of immunotherapy in stage IV non-small cell lung cancer (NSCLC) in the Netherlands”**

Christine M. Cramer – van der Welle, Marjon V. Verschueren, Merel Tonn, Bas J.M. Peters, Franz M.N.H. Schramel, Olaf H. Klungel, Harry J.M. Groen, Ewoudt M.W. van de Garde, and the Santeon NSCLC Study Group


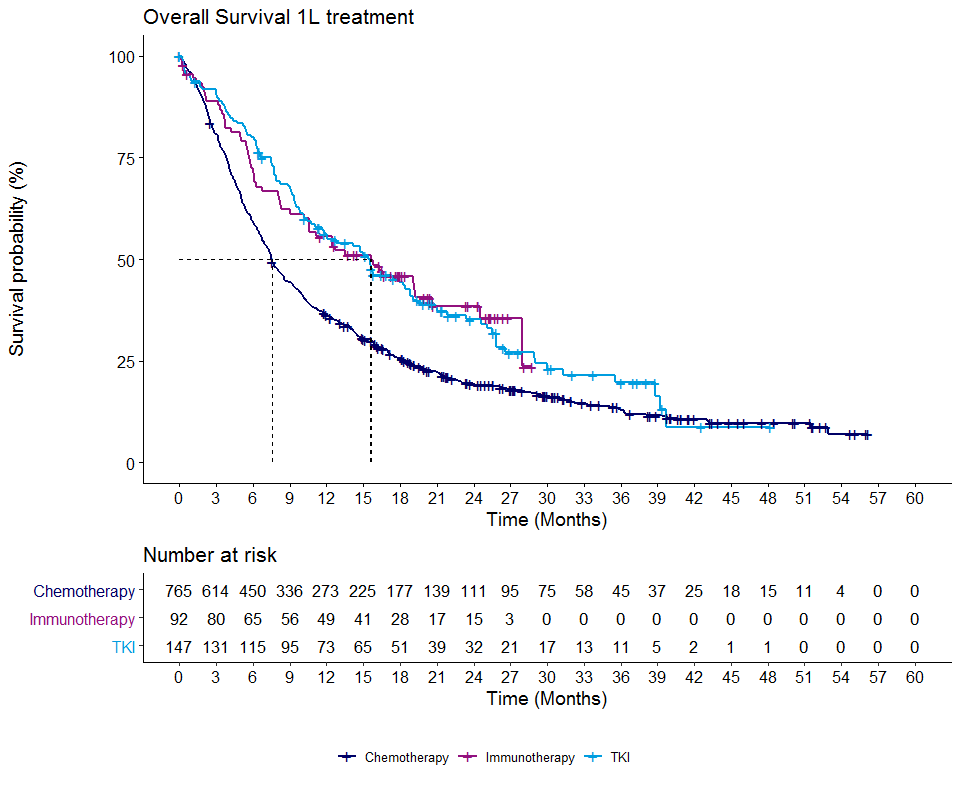


**SFigure 1**. Kaplan-Meier curves of OS in patients receiving 1L treatment in real-world
